# Impostors Dare to Compare: Associations Between the Impostor Phenomenon, Gender Typing, and Social Comparison Orientation in University Students

**DOI:** 10.3389/fpsyg.2020.01225

**Published:** 2020-06-19

**Authors:** Flora Fassl, Takuya Yanagida, Marlene Kollmayer

**Affiliations:** ^1^Department of Applied Psychology: Work, Education and Economy, Faculty of Psychology, University of Vienna, Vienna, Austria; ^2^Department of Teacher Education, Centre for Teacher Education, University of Vienna, Vienna, Austria

**Keywords:** impostor phenomenon, gender typing, femininity, masculinity, social comparison, undergraduate students, university

## Abstract

Entering university is often associated with new surroundings and challenges, which can cause distress and might result in poor mental health. A phenomenon that was shown to be linked to low self-esteem, higher stress levels, anxiety, and depression in university students is the impostor phenomenon. Impostorism is defined as a conviction to be unintelligent despite one’s academic success and was found to be closely associated with psychological femininity in previous studies. Research has also shown that people who experience higher stress levels, self-doubts, and a low self-esteem tend to engage in social comparison processes more often. Therefore, the present study aimed to explore the relationship between gender typing and impostor feelings and to investigate a possible influence of social comparison orientation on the aforementioned association. For this purpose, 278 university students (73.7% women) completed an online questionnaire. Gender typing was measured using an instrument assessing positive and negative aspects of masculinity and femininity to overcome conceptual limitations of previous studies. For social comparison processes, the general tendency to engage in social comparisons was measured. Of the participants, 8.6% experienced few, 40.3% moderate, 38.5% frequent, and 12.6% intense impostor feelings, which indicates that the impostor phenomenon is highly prevalent in university students. One of the key findings of this study concerns the association between gender typing and the impostor phenomenon. We found a moderate negative relationship between the impostor phenomenon and positive masculinity but no association with negative masculinity. In contrast, impostorism was strongly correlated with negative aspects of femininity but not at all with positive aspects of femininity. The relationship between negative femininity and impostorism was further found to be partially mediated by social comparison orientation. Social comparison orientation, however, was not found to mediate the association between positive masculinity and impostorism. This result indicates that individuals who identify with negative aspects of femininity tend to compare themselves to others more often, which is associated with stronger impostor feelings. Based on the results, we discuss possible interventions to reduce psychological distress among university students.

## Introduction

University is a place where one has to perform and where one’s academic achievement is constantly evaluated. Because of this, many students can feel pressured and consequently experience high stress levels, which can have detrimental effects on their health. As highlighted in the review by Sharp and Theiler (2018), psychological distress is a huge issue among university students worldwide and is more prevalent in university students than in the general population. Moreover, psychological stress among university students was found to be linked to problematic health behaviors, like binge drinking, heavy cigarette smoking, and self-harm. A phenomenon that is especially relevant for explaining psychological distress in university students is the impostor phenomenon.

Clance and Imes (1978), two psychotherapists who worked with high-achieving women in academica, first coined the term “impostor phenomenon” (IP) after having noticed that many of their clients experienced massive self-doubts and lived in constant fear of being exposed as intellectual impostors. The clinical symptoms that accompany the IP are “generalized anxiety, lack of self-confidence, depression, and frustration related to inability to meet self-imposed standards of achievement” (Clance and Imes, 1978, p. 242). Moreover, emotional instability, perfectionism, negative self-evaluation (Rohrmann et al., 2016), and low conscientiousness (Bernard et al., 2002) are linked to the IP. Most importantly, the IP is characterized by dysfunctional thinking patterns and attributional styles: Individuals suffering from impostor feelings are convinced to be unintelligent despite their intellectual achievements, a belief that can be explained by an inability to internalize success (Sonnak and Towell, 2001; Bernard et al., 2002). More specifically, IP sufferers explain their accomplishments by external factors, such as luck, charm, or temporary variable internal factors, e.g., excessive effort (Clance and Imes, 1978). Consequently, they experience a persistent fear that others might eventually realize that they are actually frauds and start to see them as what they really are inadequate and not intelligent enough (Leary et al., 2000). According to Clance and Imes (1978), this thinking pattern is maintained by the impostor phenomenon cycle, which is initiated when being confronted with a new task. Usually, the reaction to a new task is to either start (over)preparing immediately or to procrastinate and do the work last minute. In case of having completed the task successfully, the former strategy triggers IP sufferers to believe that they need to overexert in comparison to others (Clance et al., 1995) and that their success is only due to disproportionate effort (Leonhardt et al., 2017). The latter strategy, however, leads them to believe that they have fooled others (Clance et al., 1995), that they are not able to meet their own standards (Leonhardt et al., 2017), and that their success is due to luck (Clance and Imes, 1978). Thus, people with impostor feelings form a strong association between strain, negative emotions, and success (Clance et al., 1995). As the university context is characterized by a culture of permanent evaluation, and students frequently face new and unfamiliar tasks (Jöstl et al., 2012), it is not surprising that the IP is highly prevalent among university students. More specifically, 40–50% of students experience frequent impostor feelings (Thompson et al., 1998, 2000; Sonnak and Towell, 2001; Patzak et al., 2017).

Initially, the IP was assumed to be a distinctively female experience. Clance and Imes (1978) explained the high prevalence of IP in successful women in academia by the persisting societal gender stereotype of women not being competent or even brilliant (Broverman et al., 1972; Bian et al., 2017). They concluded that their clients had internalized this stereotype and thus, in order to protect their femininity, needed to attribute their success to external causes or temporary internal qualities instead of inherent ability. Since then, many studies have examined the link between gender and the IP. The results, however, have been equivocal. While some studies did not find any gender differences in the IP among college and university students (Cowman and Ferrari, 2002; Cokley et al., 2013), others found that women suffer more from impostor feelings than men do (King and Cooley, 1995; Kumar and Jagacinski, 2006; McGregor et al., 2008; Cokley et al., 2018). In all of these studies, gender differences were investigated by simply comparing the IP scores of women to those of men while not considering gender typing. The term gender typing (or sex typing) refers to the extent to which an individual identifies with stereotypically masculine and feminine characteristics (Kagan, 1964). Since the development of the Bem Sex-Role Inventory (Bem, 1974), femininity and masculinity are considered two independent dimensions, the former being an expressive orientation and the latter being an instrumental orientation. As gender typing is considered to be more influential than gender itself with regard to psychological functioning (Bem, 1974), it provides a high informative value for psychological phenomena like the IP. Therefore, in recent years, some studies investigating gender differences in the IP also examined the effect of gender typing (September et al., 2001; Patzak et al., 2017). However, the findings have been equivocal, too. While Patzak et al. (2017) found femininity to be related to higher IP scores and masculinity to be related to lower IP scores, September et al. (2001) found both masculinity and femininity to be associated with lower IP scores, the former with a larger effect size than the latter. Berger and Krahé (2013) revised and expanded Bem’s frequently used sex-role inventory—consisting of desirable attributes only—by including negative attributes which are seen as typical for men and women. This expansion was based on the reasonable argument that a person’s self-concept does not solely consist of positive traits. Therefore, considering only positive qualities in the assessment of gender typing might be too narrow. The newly developed questionnaire (PN-SRI, Berger and Krahé, 2013) considers four dimensions—positive femininity, negative femininity, positive masculinity, and negative masculinity—and thus enables a more comprehensive assessment of gender typing than previous instruments. So far, no studies have investigated the relationship between the IP and positive and negative facets of femininity and masculinity. In order to gain a better and more comprehensive understanding of the relationship between the IP and gender typing, the first aim of the present study is to examine this association.

Research Question 1: How do positive and negative facets of gender typing relate to the IP?

As some attributes constituting negative femininity (e.g., self-doubting or anxious) bear analogy to the symptoms IP sufferers experience (e.g., generalized anxiety or lack of self-confidence; Clance and Imes, 1978), we expect a positive relationship between negative femininity and the IP. In line with Patzak et al. (2017) and September et al. (2001), we also expect a negative association of positive masculinity and the IP. Regarding positive femininity, there are equivocal findings—one study found a small positive relationship with the IP (Patzak et al., 2017) while another study found a small negative association (September et al., 2001). For negative masculinity, no empirical findings exist, yet. Therefore, regarding the associations of the IP and negative masculinity as well as positive femininity, there is no base for proposing clear hypotheses.

In line with the impostor cycle, a recent study has shown that the working style of impostors, i.e., procrastination or over-preparation, can result in greater strain and higher stress levels (Rohrmann et al., 2016). The high stress levels people suffering from the IP experience might also lead to a higher tendency to engage in social comparison processes. Festinger (1954) coined the term “social comparison” and stated that individuals are always looking for information about themselves and, therefore, experience an innate drive for self-evaluation. In general, people preferably use objective information for self-evaluation. However, if no objective information is available, a shift of focus occurs, and the needed information is gathered by comparing one’s opinions and abilities to those of others. Even though it is still believed that social comparison is an important source of information about the self (Schneider and Schupp, 2014), additional motives for social comparison processes were identified, namely, self-improvement and self-enhancement (Gibbons and Buunk, 1999). Gibbons and Buunk (1999) modified the social comparison theory by introducing the concept of social comparison orientation (SCO) based on the assumption that individuals differ in the extent and the frequency in which they engage in social comparison processes. According to Gibbons and Buunk (1999), people with a high SCO engage in comparison processes more often and tend to be more empathetic and self-conscious, i.e., are “particularly aware of themselves” (Buunk and Gibbons, 2006, p. 18). They also revealed that SCO correlates with low self-esteem and neuroticism (Buunk and Gibbons, 2006). Moreover, Ybema and Buunk (1995) highlighted that social comparisons are facilitated by stress. One can summarize that individuals engage more often in social comparisons when they have self-doubts and a low self-esteem and are stressed (Festinger, 1954; Taylor et al., 1990; Ybema and Buunk, 1995). As the IP can be predicted by low self-esteem (Sonnak and Towell, 2001) and is usually accompanied by symptoms, such as higher stress levels, emotional instability (Rohrmann et al., 2016), and a lack of self-confidence (Clance and Imes, 1978), one can assume that people suffering from impostor feelings also tend to compare themselves more often, i.e., have a stronger social comparison orientation. This assumption is also supported by Pauline Clance’s description of a typical IP sufferer as comparing “herself to others, emphasizing their strengths and her own deficits, while minimizing weakness in others and power in herself” (Clance et al., 1995, p. 80). So far, only one study investigated the relationship between the IP and SCO. In this study, Chayer and Bouffard (2010) examined differences in SCO and their relation to the IP among 10- to 12-year-old students. Overall, the results revealed a medium correlation between SCO and the IP. In addition, Gibbons and Buunk (1999) found women to report higher levels of SCO than men. As gender typing is closely linked to gender (Wood and Eagly, 2009) and seems to be more influential than gender regarding psychological functioning (Bem, 1974), the gender difference in SCO might be explained by differences in gender typing. Moreover, some traits constituting negative femininity, e.g., self-doubts and anxiety, were already shown to facilitate social comparison processes (Festinger, 1954). Based on this, a relationship between gender typing, SCO, and the IP seems plausible. However, to our knowledge, no studies have yet investigated the relationship between these constructs. In the present study, we therefore aim to explore this possible interrelation.

Research Question 2: Is the relationship between gender typing and the IP mediated by SCO?

Gender typing is assumed to be associated not only with the IP but also with SCO as women were found to report a higher tendency to engage in social comparison (Gibbons and Buunk, 1999). Building on findings among schoolchildren (Chayer and Bouffard, 2010), SCO is expected to be related to the IP also among university students. Taken together, we assume SCO to be a mediating factor of the proposed association between gender typing and the IP in the sense that positive masculinity is associated with a lower tendency to engage in social comparisons, which is associated with lower IP scores, while negative femininity is related to higher SCO and in turn to higher IP scores.

As no study so far has investigated the associations of the IP, gender typing, and SCO among adults, the present study aimed at exploring this interrelatedness. More specifically, in a first step, we aimed at exploring how positive and negative aspects of femininity and masculinity are associated with feelings of fraudulence. Secondly, an understanding of whether SCO mediates the assumed relationship between gender typing and the IP was aimed at.

## Materials and Methods

### Participants and Procedure

The participants of the present study were recruited via groups related to universities, studies, or subjects on the social media platform Facebook. The study invitation informed possible participants about the topic of the study being social processes in everyday university life. Moreover, it stated that completing it would take approximately 15 min and that participants had a chance to win a 20€ voucher. The final sample consisted of *n* = 278 university students from Austria (82.7%) and Germany (15.1%). 2.2% of the participants indicated to study at other universities than the aforementioned. Two participants noted that this was due to them spending a semester abroad. Most of the participants were bachelor students (74.5%), followed by master students (14.4%), diploma students (10.1%), and Ph.D. students (1.1%). 73.7% of the participants were women. The age ranged from 18 to 51 years (*M* = 23.42, SD = 4.42). 94.6% of the participants were between 18 and 30 years of age. All data was collected via soscisurvey.de—a website for creating online questionnaires (Leiner, 2019). The mean duration of the study was *M* = 9.65 min (SD = 3.78). Before completing the questionnaire, the participants were asked to give their informed consent and were reminded of the voluntary nature of their participation. All scales, as well as all items, were presented in randomized order to eliminate order bias.

### Measures

#### Impostor Feelings

The German version of the Clance Impostor Phenomenon Scale (GCIPS; Klinkhammer and Saul-Soprun, 2009) was used to measure the impostor phenomenon. The GCIPS consists of 20 statements (e.g., I rarely do a project or task as well as I’d like to do it.”, “I have often succeeded on a test or task even though I was afraid that I would not do well before I undertook the task.”). Participants were asked to indicate the degree of agreement on a 5-point Likert scale ranging from 1 (*not at all true*) to 5 (*very true*). Higher scores indicate higher levels of the impostor phenomenon. The internal consistency of the GCIPS was Cronbach’s α = 0.90.

#### Gender Typing

To assess participants’ gender typing, the Positive–Negative Sex-Role Inventory (PN-SRI; Berger and Krahé, 2013) was used. The PN-SRI is composed of four subscales: the positive masculinity subscale (MAS+, e.g., “I am rational”), the negative masculinity subscale (MAS-, e.g., “I am harsh”), the positive femininity subscale (FEM+, e.g., “I am empathic”), and the negative femininity subscale (FEM-, e.g., “I am anxious”). Each of the subscales consists of six items, leading to a total of 24 items. The participants were asked to rate how characteristic each attribute is for them on a 6-point Likert scale ranging from 1 (*not at all true*) to 6 (*completely true*). The scores are calculated for each subscale. Each score indicates to what extent one ascribes oneself desirable or undesirable qualities that are considered stereotypically masculine or feminine. The internal consistencies of the four subscales were determined using Cronbach’s α: α_MAS__+_ = 0.80, α_MAS__–_ = 0.82, α_FEM__+_ = 0.87, and α_FEM__–_ = 0.77.

#### Social Comparison Orientation

The tendency to engage in social comparisons, i.e., the social comparison orientation, was assessed using the German short version of the Iowa–Netherlands Comparison Orientation Measure (INCOM; Schneider and Schupp, 2014). It consists of six items in total. Three items deal with comparing one’s abilities (e.g., “I always pay a lot of attention to how I do things compared with how others do things.”) and three with comparing one’s opinions (e.g., “I often try to find out what others think who face similar problems as I face.”). Respondents were asked to indicate their level of agreement on a 5-point Likert scale ranging from 1 (*I disagree strongly*) to 5 (*I agree strongly*). Higher scores indicate a higher tendency to engage in social comparisons. As suggested by Schneider and Schupp (2014), an overall score of the scale was computed due to high correlations between the latent constructs (*r* = 0.64, *p* < 0.001). Cronbach’s α for the German short version of the INCOM was 0.78.

### Data Analysis

In the first step, a regression model (Darlington and Hayes, 2016) was estimated to investigate relationships between positive and negative facets of gender typing and impostor feelings (Research Question 1). In the second step, we estimated a mediation model (MacKinnon and Tofighi, 2013) in order to investigate relationships between positive and negative facets of gender typing and social comparison orientation, the relationship between social comparison orientation and impostor feelings, and the indirect effects of positive and negative facets of masculinity and femininity on impostor feelings via social comparison orientation (Research Question 2).

Statistical analyses were conducted using Mplus version 8.3 (Muthén and Muthén, 1998). Model parameters were estimated using maximum likelihood method. Statistical significance of the direct and indirect effects was tested using bias-corrected bootstrapping confidence intervals based on 10,000 bootstrap draws.

## Results

### Descriptive Statistics and Preliminary Analyses

Correlation coefficients, means, and standard deviations for all variables are shown in Table 1. According to the cutoff scores recommended by Klinkhammer and Saul-Soprun (2009), 8.6% of the participants experienced few, 40.3% moderate, 38.5% frequent, and 12.6% intense impostor feelings. The overall sum score of the intensity of experienced impostor feeling was 61.20 (SD = 15.18), and the overall mean score was 3.06 (SD = 0.76). The results show that impostor feelings are negatively correlated with positive masculinity (*r* = −0.27) and positively correlated with negative femininity (*r* = 0.67) and social comparison orientation (*r* = 0.45). No statistically significant correlations were found for impostor feelings with negative masculinity and positive femininity. Social comparison orientation was positively correlated with negative femininity (*r* = 0.45). No statistically significant correlations were found for social comparison orientation with positive masculinity, negative masculinity, and positive femininity.

**TABLE 1:** Descriptive statistics and preliminary analyses: bivariate correlations, means, and standard deviations.

Variable	1	2	3	4	5	6
1. Positive masculinity	–	–	–	–	–	–
2. Negative masculinity	0.12	–	–	–	–	–
3. Positive femininity	–0.03	−**0.28**	–	–	–	–
4. Negative femininity	−**0.26**	0.09	0.08	–	–	–
5. Social comparison orientation	–0.07	0.02	0.10	**0**.**45**	–	–
6. Impostor feelings	−**0.27**	0.04	0.02	**0**.**67**	**0**.**45**	–
*M*	4.38	2.20	4.68	3.46	3.61	3.06
SD	0.85	0.90	0.88	1.02	0.81	0.76

*N = 278. Gender-typing scores range from 1 to 6, with higher values indicating stronger self-ascription of characteristics; Social Comparison Orientation Judgments range from 1 to 5, with higher scores indicating a stronger tendency to engage in social comparison processes. Impostor feelings scores range from 1 to 5, with higher scores indicating higher levels of impostor feelings. Statistically significant results at α = 0.05 are in boldface.*

### Gender Typing and IP

The results of the regression model indicate a negative relationship between positive masculinity and impostor feelings ($\hat{\beta }=-0.09$, 95% CI [−0.17, 0.00]) and a positive relationship between negative femininity and impostor feelings ($\hat{\beta }=0.49$, 95% CI [0.42, 0.56]) while statistically controlling for all other facets of femininity and masculinity (Research Question 1). Negative masculinity and positive femininity, however, are not related to impostor feelings. The regression model explained 46.3% of the variance in impostor feelings (see Table 2).

**TABLE 2:** Regression model results: relationship between positive and negative facets of masculinity and femininity and impostor feelings.

Model	Est. (SE)	95% CI	Std. Est.
Positive masculinity	**−0.09** (0.04)	[−0.17, 0.00]	−0.10
Negative masculinity	−0.02 (0.04)	[−0.10, 0.06]	−0.03
Positive femininity	−0.04 (0.04)	[−0.12, 0.04]	−0.05
Negative femininity	**0.49** (0.04)	[0.42, 0.56]	0.65

*N = 278; *R*^2^ = 0.46; 95% CI = 95% confidence interval. Statistically significant results at α = 0.05 are in boldface.*

### Gender Typing, SCO, and Impostor Feelings

**FIGURE 1:**
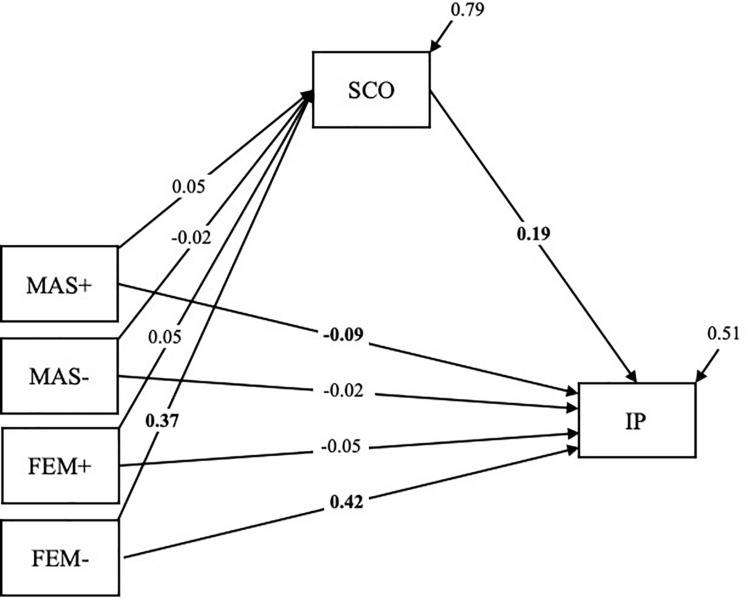
Relationship between positive and negative facets of masculinity and femininity and impostor feelings mediated by social comparison orientation. MAS+ = positive masculinity; MAS- = negative masculinity; FEM+ = positive femininity; FEM- = negative femininity; SCO = social comparison orientation; IP = impostor feelings. Unstandardized estimates. Statistically significant results at α = 0.05 are in boldface.

The mediation model shows a positive relationship between negative femininity and social comparison orientation ($\hat{\beta }=0.37$, 95% CI [0.27, 0.46]) while statistically controlling for all other facets of gender typing. All other facets of gender typing are not related to social comparison orientation. As expected, the results reveal a positive relationship between social comparison orientation and impostor feelings ($\hat{\beta }=0.19$, 95% CI [0.09, 0.28]). As a result, an indirect effect was found for negative femininity on impostor feelings via social comparison orientation ($\hat{\beta }=0.07$, 95% CI [0.03, 0.12]) while statistically controlling for all other effects (Research Question 2). Indirect effects for all other components of gender typing were statistically not significant. The direct effect of negative femininity on impostor feelings controlling for the indirect effect was statistically significant ($\hat{\beta }=0.42$, 95% CI [0.35, 0.50]) indicating a partial mediation effect. The mediation model explained 20.7% of the variance in social comparison orientation and 49.4% of the variance in impostor feelings (see Table 3 and Figure 1).

**TABLE 3:** Mediation model results: relationship between positive and negative facets of masculinity and femininity and impostor feelings mediated by social comparison orientation.

Independent variable	Mediating variable	Dependent variable	Est. (SE)	95% CI	Std. Est.
**Individual components of the indirect effect**					
MAS+	SCO	–	0.05 (0.06)	[−0.06, 0.17]	0.05
MAS-	SCO	–	−0.02(−0.05)	[−0.12, 0.09]	–0.02
FEM+	SCO	–	0.05 (0.05)	[−0.04, 0.16]	0.06
FEM-	SCO	–	**0.37**(0.05)	[0.27, 0.46]	0.46
–	SCO	IP	**0.19**(0.05)	[0.09, 0.28]	0.20
**Indirect effect**					
MAS+	SCO	IP	0.01 (0.01)	[−0.01, 0.04]	0.01
MAS-	SCO	IP	0.00 (0.01)	[−0.03, 0.02]	0.00
FEM+	SCO	IP	0.01 (0.01)	[−0.01, 0.03]	0.01
FEM-	SCO	IP	**0.07**(0.02)	[0.03, 0.12]	0.09
**Direct effect controlling for the indirect effect**					
MAS+	–	IP	−**0.09**(0.04)	[−0.18, -0.02]	–0.11
MAS-	–	IP	−0.02(0.04)	[−0.10, 0.06]	–0.02
FEM+	–	IP	−0.05(0.04)	[−0.13, 0.03]	–0.06
FEM-	–	IP	**0.42**(0.05)	[0.35, 0.50]	0.56

*N = 278; *R*^2^_*SCO*_ = 0.21; *R*^2^_*IP*_ = 0.49; MAS+ = positive masculinity; MAS- = negative masculinity; FEM+ = positive femininity; FEM- = negative femininity; SCO = social comparison orientation; IP = impostor feelings; Est. = unstandardized parameter estimate; SE = standard error; 95% CI = 95% bias-corrected bootstrap confidence interval; std. est. = standardized estimate. Statistically significant results at α = 0.05 are in boldface.*

## Discussion

The present study aimed at gaining insights into the interplay of the IP, gender typing, and SCO. In the past, each of the aforementioned constructs has been studied intensely separately. However, previous studies have not investigated their interrelationship, yet. Aligning with prior research, the current study confirms that impostor feelings are highly prevalent among university students as only 8.6% of the participants experienced few impostor feelings while 51.1% experienced frequent or intense impostor feelings. This becomes even more apparent when looking at the overall mean score of the IP, which indicates that on average our sample experienced frequent impostor feelings (Klinkhammer and Saul-Soprun, 2009). This finding is relevant for research on distress among university students since the IP might explain many aspects that have been studied in this context: perfectionism, anxiousness, unfavorable goal orientations, or dysfunctional attribution types. Thus, constructs related to impostor feelings might be productive starting points of interventions for distress in a student population. Therefore, future research investigating distress in a university context should consider the experience of impostor feelings as a possible explanation.

Our first research question concerned the association of gender typing and the IP. Although gender differences in the IP often have been reported, only few studies have investigated the role of gender typing for impostorism (September et al., 2001; Patzak et al., 2017). Gender typing is conceptualized as being more informative than gender with regard to explaining psychological phenomena (Bem, 1974) and studies investigating its relationship to the IP found that impostor feelings are better explained by gender typing than gender itself (Patzak et al., 2017). More specifically, it was found that people with low masculinity are likely to experience impostor feelings whereas people with high masculinity are not. Previous studies assessed gender typing using instruments that only considered positive facets of masculinity and femininity. However, notions of femininity and masculinity are based not only on positive traits but also on negative ones (Berger and Krahé, 2013). Therefore, in this study, we reexamined the established relationship between gender typing and the IP using an instrument that measures positive and negative aspects of gender typing. In line with Patzak et al. (2017), we found gender typing to explain more than 46% of the variance in impostor feelings. Moreover, like previous studies (September et al., 2001; Patzak et al., 2017), we found a moderate negative relationship between positive masculinity and impostorism indicating that the more students attribute positive aspects of masculinity (e.g., practical, rational) to themselves, the less they suffer from the IP. In contrast, negative masculinity (e.g., arrogant, inconsiderate) was not found to be associated with impostor feelings. As expected, the results indicated a strong positive relationship between negative femininity (e.g., anxious, disoriented) and impostor feelings. Interestingly, however, no significant relationship was found between positive femininity (e.g., emotional, sensitive) and the IP. This contradicts previous findings which indicated that a self-ascription of femininity relates to stronger impostor feelings (Patzak et al., 2017). The dimensions usually seen as stereotypically feminine are communion, warmth, or expressivity (Kite et al., 2008) and are depicted in positive femininity. Thus, the current data leads us to believe that impostor feelings are not per se related to feminine gender typing but rather to the self-ascription of attributes constituting negative femininity. Interestingly, the characteristics of negative femininity closely resemble some facets of neuroticism, i.e., anxiety, depression, self-consciousness, and vulnerability (Costa and McCrae, 1992). Thus, it would be worth investigating the relationship between gender typing and neuroticism and its association to the impostor phenomenon in future studies. Another aspect worth exploring is the analysis of the PNSRI scores within Bem’s (1974) gender typology. Bem’s gender typology consists of feminine, masculine, and androgyne gender types that are created from scores of the two BSRI scales. This characterization is of relevance with regard to the impostor phenomenon as it was shown that androgynous people experience feelings of fraudulence less frequently (September et al., 2001; Patzak et al., 2017). Therefore, it can be assumed that androgyny functions as a resilience factor against the IP. As a person’s self-concept consists of not only positive but also negative aspects (Berger and Krahé, 2013), it might be interesting to advance Bem’s typology by also including negative aspects of gender typology. Analyzing scores from the PNSRI (Berger and Krahé, 2013) by characterizing them within Bem’s typology would most probably result in numerous gender types. It would be very interesting to explore what gender types can be found and how these relate to various constructs. Moreover, an established typology might be extremely beneficial with regard to the exploration of possible protective and risk factors for the impostor phenomenon. Such an endeavor is of relevance as there have been contradictive research findings on risk factors so far. As the impostor phenomenon can be conceptualized as a self-destructive thought pattern which in turn might result in or be linked to self-destructive behavior, it is surprising that earlier studies found masculinity to be a factor that predisposes individuals to self-destructive patterns (Tsirigotis et al., 2013). Contrary to this finding and in line with prior studies (September et al., 2001; Patzak et al., 2017), we found positive masculinity to be negatively related to the IP, which might in turn point toward it being a resilience factor. Altogether, our study shows that disentangling positive and negative aspects of gender identity is crucial for explaining impostor feelings in men and women.

Our second research question concerned the interrelatedness of gender typing, IP, and SCO. We anticipated the relationship between gender typing and the IP to be mediated by the tendency to engage in social comparison processes. This hypothesis was based on three findings. First, there is a close similarity between the symptoms of the IP and characteristics that were shown to facilitate social comparison processes (Festinger, 1954; Taylor et al., 1990; Ybema and Buunk, 1995). Second, schoolchildren experiencing impostor feelings were found tend to compare themselves more often (Chayer and Bouffard, 2010). Third, women were found to have higher tendency to engage in social comparison processes than men (Gibbons and Buunk, 1999). Contrary to our expectation, the data suggests that SCO only mediates the association between negative femininity and IP but not the association between positive masculinity and the IP. The experience of strong impostor feelings by people who ascribe themselves stereotypically negative feminine characteristics can thus partly be explained by a higher tendency to engage in social comparison processes. This indicates that negative femininity is directly and indirectly (via SCO) associated with stronger IP, while positive masculinity is only directly associated with lower IP. The positive relationship between negative femininity and SCO can be explained in two ways: first, self-doubts are one characteristic of negative femininity and were shown to facilitate engagement in social comparison processes (Festinger, 1954). Second, as stated before, negative femininity also bears some similarity to neuroticism which was found to be linked to a higher SCO (Van der Zee et al., 1998).

The association between the IP and SCO suggested by the mediation model indicates that SCO is indeed an important antecedent of the IP. Since this relationship has only been studied once among schoolchildren (Chayer and Bouffard, 2010), more research is needed. To obtain a clearer picture on the constructs, future research could explore the relationship of these within a longitudinal design and predominantly focus on the influence of the developmental factor on the constructs and their associations. Inspired by Chayer and Bouffard (2010), it might also be of interest to also investigate the different social comparison preferences of people suffering from impostor feelings, i.e., whether they tend to engage in upward or downward comparison processes and whether they contrast or identify themselves with the comparison target. This is of importance as different social comparison preferences were shown to evoke different emotions (Smith, 2000). In their study, Chayer and Bouffard (2010) found a positive correlation between impostor feelings and upward contrast and downward identification. Since the sample consisted of children with mostly weak impostor feelings, more research is needed to establish a clearer picture on the social comparison preferences of people suffering from impostor feelings.

As impostor feelings are experienced quite frequently by students and were found to be associated with depression (McGregor et al., 2008), it is highly relevant to take measures to reduce the IP in university students. Based on our findings, we propose that reducing negative femininity or SCO might be beneficial to university students due to their close relationship to the IP. Prior research found lower levels of self-compassion to be associate with higher levels of IP (Patzak et al., 2017). Therefore, interventions aiming at increasing self-compassion might be effective in fighting feelings of fraudulence. When trying to combat impostor feelings in a university context, a focus on SCO might be especially effective as measures can be taken from both teachers and students themselves. Overall, we believe that interventions that strengthen the focus on the self and reduce competition between students might help to reduce social comparison processes. In assessing students’ performances, a shift from an “ability game” to an “equity game” might be beneficial as it does not foster comparisons among students but reaffirms hard work, persistence, and self-improvement (Covington, 2000). An equity game is characterized by criteria-referenced assessment and formative feedback which might further strengthen the students’ focus on their own competences and improvement. Since people were shown to engage in social comparison processes when stressed (Ybema and Buunk, 1995), measures of stress reduction might also be effective in reducing impostor feelings in university students. Social support has been found to moderate the impact of stress (Cobb, 1976). Therefore, classes that enable and promote group work and, therefore, foster network building among students might be helpful to reduce SCO. In addition, measures to reduce impostor feelings are not restricted to teachers but can be applied by students experiencing such feelings themselves. Keeping a reflective diary might help students to identify self-destructive thoughts and reduce stress. A study has shown that students who keep reflective diaries develop explanations for their feelings of stress and an improved self-efficacy with regard to future stressful situations (Travers, 2011). Moreover, the participants also experienced higher self-acceptance after keeping a diary. Therefore, diary keeping can be an effective measure to raise the students’ awareness that academia is only one part of their lives and consequently might reduce the assigned importance.

## Limitations

The present study has several strengths, but some limitations need to be considered. Firstly, the present study was a correlation study and thus no causal conclusions can be made. Secondly, although the impostor phenomenon is defined by feelings of intellectual phoniness despite academic and/or professional success (Clance and Imes, 1978), in the present study, the IP was measured only subjectively using the GCIPS (Klinkhammer and Saul-Soprun, 2009). Therefore, it would be reasonable to include objective measurements of success (e.g., grade average) in future studies to see if different results occur between high-achievers and students with average or low achievement. Thirdly, the sample of the present study mainly consisted of female undergraduate university students that were recruited via Facebook. In order to gain a better understanding, future research should study the relation of the impostor phenomenon, social comparison, and gender typing separately in men and women. Moreover, in the recruitment process, future studies should consider different channels and not solely focus on platform users in order to yield more representative results. As university students represent an intensely studied sample, it would be interesting to investigate the discussed interplay in a sample consisting only of working people or Ph.D. students. The latter might suffer even more from impostorism due to them experiencing more pressure to be successful than undergraduate university students. Thirdly, the items measuring social comparison orientation were not specifically coined to suit situations university students encounter. It would be interesting to replicate the study using an instrument that operationalizes comparison processes specifically among university students.

## Conclusion

The present study contributes to the literature on psychological distress in university students by examining antecedents of the impostor phenomenon, i.e., the conviction to be unintelligent despite one’s academic success. Once again, the findings of the present study highlight the high prevalence of the IP among university students with 51.1% of the participants experiencing frequent to intense impostor feelings. The results show that the more university students identify with negative aspects of femininity and the less they identify with positive aspects of masculinity, the more they experience impostor feelings. Interestingly, positive masculinity had only a direct effect on the IP, while the relationship between negative femininity and the IP was partially mediated by social comparison orientation. In sum, the study demonstrates that universities should avoid fostering competition and facilitating comparisons among students but rather focus on the students’ individual talents, diligence, and self-improvement.

## Data Availability Statement

The datasets generated for this study are available on request to the corresponding author.

## Ethics Statement

Ethical review and approval was not required for the study on human participants in accordance with the local legislation and institutional requirements. The patients/participants provided their written informed consent to participate in this study.

## Author Contributions

All listed authors contributed meaningfully to the manuscript, contributed to the study design and analyzed or interpreted the data, approved the final version of the manuscript for submission. FF and MK developed the study concept. FF and TY prepared the draft manuscript, and MK provided critical revisions.

## Conflict of Interest

The authors declare that the research was conducted in the absence of any commercial or financial relationships that could be construed as a potential conflict of interest.
